# Clinical Clerkships in General Medicine Enable Students to Acquire Basic Medical Competencies and Experience in Community-Based Integrated Care: A Descriptive Questionnaire-Based Study

**DOI:** 10.7759/cureus.36495

**Published:** 2023-03-21

**Authors:** Masaki Tago, Risa Hirata, Kiyoshi Shikino, Takashi Watari, Shun Yamashita, Yoshinori Tokushima, Midori Tokushima, Hidetoshi Aihara, Naoko E Katsuki, Shu-ichi Yamashita

**Affiliations:** 1 Department of General Medicine, Saga University Hospital, Saga, JPN; 2 Department of General Medicine, Chiba University Hospital, Chiba, JPN; 3 General Medicine Center, Shimane University Hospital, Izumo, JPN

**Keywords:** national model core curriculum for undergraduate medical education, university, specialty program, medical education, general medicine, clinical clerkship

## Abstract

Background

No previous research has targeted educators regarding educational practice and the achievements of students in terms of the learning objectives of clinical clerkships in university general medicine departments of Japan. We aimed to clarify the characteristics of clinical clerkships in Japanese general medicine departments using a questionnaire administered to chairpersons of university general medicine departments.

Methods

This was a descriptive questionnaire-based study using Google Forms (Google, Inc., Mountain View, CA, USA). We asked the chairpersons of general medicine departments in Japanese universities the following questions, with responses given on a 5-point Likert scale: Question 1: How well are primary symptoms in the national model core curriculum for undergraduate medical education taught in clinical clerkships in university general medicine departments? Question 2: How successfully can students achieve the learning objectives of the national model core curriculum for undergraduate medical education through clinical clerkships in general medicine departments of university hospitals? Question 3: How successfully can students achieve the learning objectives of the national model core curriculum for undergraduate medical education through clinical clerkships in other community clinics or hospitals? The results of the questionnaire responses are described as mean±standard deviation.

Results

Of the 71 Japanese universities with general medicine departments, 43 were included in the analysis. For Question 1, the symptoms and pathophysiologies with a mean score of 4 points or higher were fever, general malaise, anorexia, weight loss or gain, edema, abdominal pain, lymphadenopathy, and headache. All those symptoms require basic medical competencies. For Questions 2 and 3, the intramural clinical clerkship of general medicine departments had a higher mean score than the extramural clinical clerkship for diagnostic reasoning that emphasizes medical history and physical examination and a comprehensive approach to patients with multiple health problems. In contrast, the extramural clinical clerkship, in which medical students can build experience with community-integrated care, had a mean score of 3 points or higher for all items.

Conclusions

The clinical clerkship in general medicine departments of Japanese universities provides students with chances to acquire clinical competencies regarding primary symptoms and pathophysiologies. Additionally, the extramural clinical clerkship provides experience in community-based integrated care, including home medical care, collaboration, health and welfare, and long-term care.

## Introduction

Japan is the most super-aged society in the world, with 28.8% of the population aged 65 years and older [1,2]. Therefore, it is important to train physicians who can effectively manage limited healthcare resources and provide community-based integrated care based on an understanding of the physical and mental changes in older adults, patients with comorbidities and multiple problems, and the long-term care and welfare systems [3]. The national model core curriculum for undergraduate medical education (hereinafter, the model core curriculum) was first published in 2001 and has since been revised to meet the needs of an aged society and the global standardization of medical education [4,5]. The model core curriculum revised in 2016 clearly states that its objective is to cultivate physicians who can respond to the diverse needs of society [3]. The model core curriculum focuses on the acquisition of basic clinical competencies such as knowledge of an approach based on symptoms and pathophysiology, medical interviewing and physical examination skills, and a professional attitude and behavior appropriate for a physician. Training in various and diverse topics involving basic medical knowledge, skills, and attitudes is crucial, rather than focusing solely on specialized knowledge, in particular medical fields [3]. Furthermore, a hands-on clinical clerkship is required to maintain the quality of medical education at an international level and improve and develop medical education nationally [3,6]. In Japan, a shift from teacher-centered medical education that is based on knowledge transfer to learner-centered, hands-on medical education has been attempted since the 1990s [7,8]. Today, with the decision to implement objective, structured clinical examination and the legalization and introduction of student doctors [9,10], the clinical clerkship has gradually shifted to a practice-based training program.

Japanese medical students can attend new outpatients in the general medicine department at a university, where they can practice the sequence of the medical interview, physical examination, laboratory or imaging tests, diagnosis, and treatment. In this way, students learn the importance and difficulty of these processes and the importance of the physician-patient relationship and communication [11,12]. Furthermore, many medical universities in Japan conduct extramural community healthcare training in which they participate in clinical clerkships at community hospitals, clinics that include home medical care support, and long-term care facilities. Through community healthcare training, medical students can learn about relationships with home care nurses, long-term care workers, care managers, and other professionals, as well as comprehensive patient-centered care in the communities where patients live [13]. Japanese general physicians require different skills depending on their workplace setting [14], but diagnostic reasoning skills comprise the bulk of their necessary skills [15]. Therefore, a clinical clerkship in general medicine departments at universities and in the community is highly likely to be beneficial for medical students because they can improve their basic clinical competencies such as medical interviewing, physical examination skills, providing explanations to patients and communication skills, planning of medical treatment, and interpretation of examination results [16,17].

Most studies on the actual state of medical education in university hospital general medicine departments are questionnaire-based studies conducted among students before and after training or studies regarding issues of community medicine training and the skills students can acquire [11,18-20]. To the best of our knowledge, no previous study has been conducted among educators regarding the educational conditions of clinical clerkships in general medicine departments at universities and the achievement of the learning objectives of clinical clerkships by medical students. In this study, we aimed to clarify the characteristics of clinical clerkships in university general medicine departments by querying educators regarding how well medical students are taught about symptoms and pathophysiologies in clinical clerkships in general medicine departments at Japanese universities and how well students achieve the learning objectives in intramural and extramural clinical clerkships within general medicine departments.

## Materials and methods

This descriptive study was a part of the nationwide questionnaire-based study of general medicine departments at university hospitals in Japan belonging to the Council of Japanese University Hospitals for General Medicine [21,22]. The council covers general medicine departments in university hospitals throughout Japan, and the aim of its annual meeting is to promote communication and information-sharing among Japanese university hospitals. We conducted an Internet-based survey of 82 universities across the country on June 28, 2021, using e-mail and Google Forms (Google, Inc., Mountain View, CA, USA). The person responsible for completing the questionnaire was the chairperson of the general medicine department in the main hospitals of each university. Each survey respondent was asked to state the name of their department and their position. We excluded affiliated university hospitals from this study because the scale and roles of Japanese affiliated university hospitals are diverse, and it is impossible to bring them together as a single group. Additionally, surveys with incomplete responses were excluded from the analysis.

In the survey, we asked which symptoms and pathophysiologies are taught and how successfully medical students achieve the learning objectives of clinical clerkships in general medicine departments inside and outside the university hospital. The survey comprised the following questions, with responses given on a 5-point Likert scale (even ordinal scale of 1-5, 1: not at all achievable to 5: fairly achievable, no legend for 2, 3, and 4): Question 1: How well are primary symptoms in the national model core curriculum for undergraduate medical education taught in clinical clerkships at university general medicine departments? Question 2: How successfully can students achieve the learning objectives of the model core curriculum through clinical clerkship general medicine departments of university hospitals? Question 3: How successfully can students achieve the learning objectives of the model core curriculum through clinical clerkship in other community clinics or hospitals?

The questionnaire was created, tested, and validated in a pilot trial by the authors, all of whom were assistant professors or above and were involved in teaching general medicine at their universities. The results of the questionnaire responses are described as mean±standard deviation (SD). The Statistical Package for the Social Sciences (SPSS) version 25 (IBM SPSS Statistics, Armonk, NY, USA) was used for statistical analyses.

Information regarding this study was provided on the first page of the questionnaire in Google Forms. All respondents gave their informed consent on the questionnaire website. This study involved neither human subjects nor personal information, and the university names were anonymized for the analysis. For these reasons, the Ethics Committee of Saga University Hospital waived ethics approval for this study. All methods were carried out in accordance with relevant guidelines and regulations.

## Results

**Figure 1:**
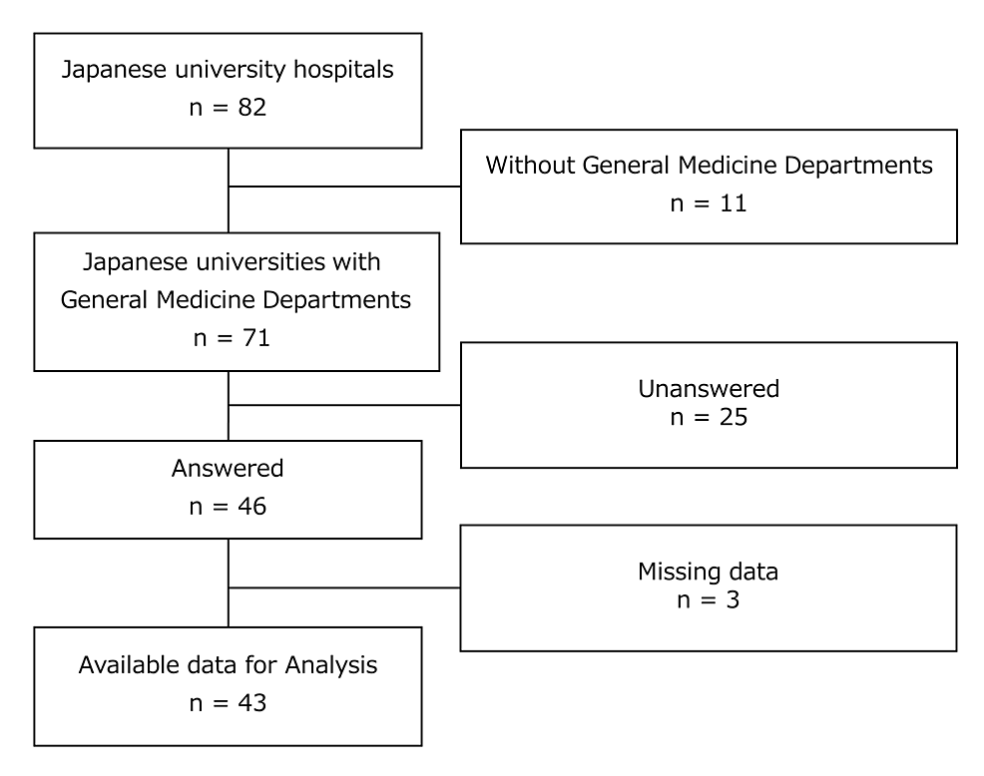
Data flow diagram

Among all Japanese university hospitals, the chairperson of 46/71 (64.7%) universities with general medicine departments responded to the questionnaire. After excluding three surveys with missing data, we included responses from 43 universities in the analysis (Figure 1). The results of responses to Question 1 are shown in Table 1. In the responses to Question 1, the symptoms and pathophysiologies with a mean score of 4 points or higher were fever, general malaise, anorexia, weight loss/gain, edema, abdominal pain, lymphadenopathy, and headache. The symptoms and pathophysiologies with a mean of less than 3 points were shock, cardiac arrest, convulsion, jaundice, menstrual abnormality, and trauma/burn. Of the 37 symptoms and pathophysiologies, 22 (59.5%) were scored as 3.5 points by more than half of the university chairpersons.

**Table 1:** Symptoms and pathophysiology taught during clinical clerkships in the general medicine department of university hospitals Range: 1 (not at all achievable) to 5 (fairly achievable) SD: standard deviation

Symptoms and pathophysiology	Overall (mean±SD) (n=43)	Number of universities that responded (4 or 5) (number (%)) (n=43)
Fever	4.3±0.9	35 (81.4)
General malaise	4.1±0.9	32 (74.4)
Anorexia	4.1±0.9	31 (72.1)
Weight loss/gain	4.0±0.9	29 (67.4)
Shock	2.6±1.2	9 (20.9)
Cardiac arrest	2.2±1.3	7 (16.3)
Consciousness disorder/syncope	3.5±1.1	22 (51.2)
Convulsion	2.8±1.2	12 (27.9)
Vertigo and dizziness	3.9±1.0	27 (62.8)
Dehydration	3.7±1.0	22 (51.2)
Edema	4.1±0.8	32 (74.4)
Rash	3.2±1.1	13 (30.2)
Cough/sputum	3.8±0.9	24 (55.8)
Bloody sputum/hemoptysis	3.1±0.9	9 (20.9)
Dyspnea	3.7±1.0	27 (62.8)
Chest pain	3.9±0.9	29 (67.4)
Palpitations	3.8±0.9	24 (55.8)
Pleural effusion	3.2±1.0	15 (34.9)
Dysphagia	3.4±1.0	17 (39.5)
Abdominal pain	4.0±0.9	29 (67.4)
Nausea/vomiting	3.8±1.0	25 (58.1)
Hematemesis/melena	3.0±1.0	13 (30.2)
Constipation/diarrhea	3.7±0.9	24 (55.8)
Jaundice	2.9±0.9	7 (16.3)
Abdominal distension (including ascites)/mass	3.4±0.9	15 (34.9)
Anemia	3.9±0.9	26 (60.5)
Lymphadenopathy	4.0±0.9	30 (69.8)
Urine volume/urination abnormality	3.4±1.0	14 (32.6)
Hematuria/proteinuria	3.3±0.9	13 (30.2)
Menstrual abnormality	2.5±1.0	4 (9.3)
Anxiety/depression	3.7±1.1	26 (60.5)
Amnesia	3.4±1.1	20 (46.5)
Headache	4.0±0.9	30 (69.8)
Motor paralysis/muscle weakness	3.5±1.0	17 (39.5)
Low back pain	3.9±1.0	27 (62.8)
Arthralgia/joint swelling	3.8±0.9	26 (60.5)
Trauma/burn	2.1±1.0	3 (7.0)

The results of responses to Questions 2 and 3 are shown in Table 2. The only learning objective with a score of 4 points or higher in both intramural and extramural clinical clerkships in general medicine departments was diagnostic reasoning that emphasizes medical history/physical examination. Intramural training had higher mean scores than extramural training for diagnostic reasoning that emphasizes medical history/physical examination and care using a comprehensive approach for patients with multiple health problems. However, extramural training had a mean score of 3 points or higher for all learning objectives.

**Table 2:** Achievement of learning objectives of the intramural and extramural general medicine clerkship Range: 1 (not at all achievable) to 5 (fairly achievable) SD: standard deviation

Learning objectives	Mean±SD (n=43)
Intramural clinical clerkship	
Assemble or follow diagnostic reasoning that emphasizes medical history/physical examination (including cases without a diagnosis)	4.1±0.9
Experience a comprehensive approach to patients with multiple health problems	3.8±0.8
Have a viewpoint of family and community and participate to the extent possible in medical practice with more consideration for psychological/social background	3.2±1.1
Experience home medical care	2.0±1.3
Experience interprofessional work and recognize its importance	2.9±1.1
Refer to the health, medical, welfare, and long-term care systems in the clinical settings	2.8±1.1
Extramural clinical clerkship	
Assemble or follow diagnostic reasoning that emphasizes medical history/physical examination (including cases without a diagnosis)	3.5±1.3
Experience a comprehensive approach to patients with multiple health problems	3.6±1.3
Have a viewpoint of family and community and participate to the extent possible in medical practice with more consideration for psychological/social background	3.6±1.2
Experience home medical care	3.6±1.3
Experience interprofessional work and recognize its importance	3.5±1.2
Refer to the health, medical, welfare, and long-term care systems in the clinical settings	3.4±1.2

## Discussion

The objective of this study was to investigate the educational situation regarding symptoms and pathophysiology in clinical clerkships at university general medicine departments and how successfully medical students achieve the related learning objectives, both intramurally and extramurally, to clarify the characteristics of clinical clerkships in general medicine departments at Japanese universities. The study findings showed that more than half of the university general medicine departments included in our survey covered 59.5% of the essential symptoms and pathophysiologies listed in the model core curriculum. Furthermore, the combination of extramural clinical clerkships enables students to achieve all the learning objectives of clinical clerkships in general medicine departments according to the model core curriculum.

Generalists often treat patients with various chief complaints and symptoms, including fever, general malaise, anorexia, weight loss or gain, edema, abdominal pain, lymphadenopathy, and headache [23]. These items were all listed in Question 1 of our survey, with scores of 4 points or higher being satisfactory. Generalists are required to provide a wide range of cross-organ care [24], covering common diseases to diagnostically challenging cases, and they must be able to comprehensively cope with biological, psychological, and social issues. Therefore, clinical reasoning is essential to treat patients’ problems according to their medical history and physical findings and to infer the correct pathophysiology and disease for a correct diagnosis. Generalists, in particular, are experts in diagnostic reasoning, in which they must engage in their daily clinical practice [15]. Additionally, although medical students can build experience with diseases specific to each organ in clinical clerkships within specialty departments subdivided by organ, many symptoms and diseases listed in the model core curriculum will not be encountered in such settings. Clinical clerkships in general medicine departments enable students to build experience with a wide range of patient symptoms and diseases that cannot be obtained in specialty departments, thereby complementing their training experience.

In Question 1 of our survey, shock, cardiac arrest, trauma or burn, convulsion, menstrual abnormality, and jaundice had scores of less than 3 points. Most general medicine departments in Japanese universities focus primarily on outpatient care, and this outpatient care may be focused on general internal medicine and diagnostic medicine [25]. Because patients with trauma and burns who visit a university hospital are likely to be in severe condition and need hospitalization, there are few opportunities to gain experience with such conditions in a university general medicine department that is focused on internal medicine. Therefore, it is difficult to provide training in treating such patients as part of clinical clerkships. However, generalists are expected to be able to manage critical patients [14,26]. Thus, the general medicine department of a university should provide practice opportunities within a wide range of intensive care specialties, mainly internal medicine, and should train students in providing emergency and inpatient care within the hospital. Menstrual abnormality is likely to be a problem in younger women, who are more likely to be hesitant about visiting a hospital and prefer to see a gynecologist directly [27,28]. Hepatologists or gastroenterologists may treat jaundice directly in a patient with a severe condition such as scleral jaundice, externally visible abnormalities, or serum bilirubin 3 mg/dL (51 µmol/L) or higher [29]. Accordingly, general medicine, emergency medicine, and other organ-specific specialty departments should provide complementary clinical clerkships to cover these symptoms and pathophysiologies with which students require experience.

In Question 2 of our study questionnaire, diagnostic reasoning that emphasizes medical history and physical examination scored more than 4 points; generalists must cultivate high-level skills in medical interviewing, physical examination, and diagnostic reasoning among students [14-16,30]. University hospitals should focus on patients with multiple problems or difficult diagnoses [31,32]. Therefore, more time should be spent on diagnostic reasoning emphasizing medical history and physical examination, particularly because outpatients spend more time at university hospitals than at hospitals outside a university [33]. Consequently, students can successfully learn diagnostic reasoning in clinical clerkships within general medicine departments of university hospitals.

The achievement level for extramural training in Question 2 of our survey was 3 points or higher in all categories, including home medical care, interprofessional work, and health, medical, welfare, and long-term care systems; these items scored less than 3 points for intramural training. In Japan, the Community Health Care Vision has been promoted as a government policy to provide high-quality and efficient healthcare to cope with changes in healthcare needs owing to depopulation and a super-aged population [34]. Under these circumstances, university hospitals are expected to mainly provide high-level acute care, making it impossible for students at university hospitals to be exposed to common diseases and chronically ill patients requiring long-term care. Although students cannot gain experience in critical care during extramural training, which is one of the learning objectives of a clinical clerkship, they can build experience with community-integrated care, including home medical care and cooperation among nursing care, welfare, and healthcare services [35]. Thus, extramural training in general medicine is considered to provide students with a well-balanced education and complement the experience that they would lack if they only trained at university hospitals.

This study had several limitations. First, this was a questionnaire survey, and 25 universities (35.2%) did not respond to our survey. Second, we could not verify the external validity of our study findings because the questionnaire was designed only for general medicine departments in Japanese universities and was based on the model core curriculum for medical education in Japan. Third, because this was a descriptive study regarding clinical clerkship education in university general medicine departments, we could not assess its effectiveness or impact. Fourth, this study targeted the chairpersons of general medicine departments rather than medical students receiving education, which can fail to reflect the actual situation of clinical clerkship. Further research is required to clarify these points.

## Conclusions

The clinical clerkship in general medicine departments of Japanese universities provides students with chances to acquire clinical competencies regarding primary symptoms and pathophysiologies. Additionally, an extramural clinical clerkship provides students with experience in community-based integrated care, including home medical care, collaboration, health and welfare, and long-term care.
